# Concurrent MEK targeted therapy prevents MAPK pathway reactivation during BRAF^V600E^ targeted inhibition in a novel syngeneic murine glioma model

**DOI:** 10.18632/oncotarget.12419

**Published:** 2016-10-03

**Authors:** Stefan Grossauer, Katharina Koeck, Nicole E. Murphy, Ian D. Meyers, Mathieu Daynac, Nathalene Truffaux, Albert Y. Truong, Theodore P. Nicolaides, Martin McMahon, Mitchel S. Berger, Joanna J. Phillips, David C. James, Claudia K. Petritsch

**Affiliations:** ^1^ Department of Neurological Surgery, Brain Tumor Research Center, San Francisco, CA 94158, USA; ^2^ Department of Pediatrics, University of California San Francisco, San Francisco, CA 94158, USA; ^3^ Huntsman Cancer Institute, University of Utah, Salt Lake City, UT 84112, USA; ^4^ Department of Neurological Surgery, Feinberg School of Medicine, Northwestern University, Chicago, IL 60611, USA; ^5^ Helen Diller Comprehensive Cancer Research Center, University of California San Francisco, San Francisco, CA 94158, USA; ^6^ Eli and Edythe Broad Center of Regeneration Medicine and Stem Cell Research, University of California San Francisco, San Francisco, CA 94158, USA

**Keywords:** MAPK pathway reactivation, dabrafenib, primary adaptive therapy resistance, syngeneic high-grade astrocytoma model

## Abstract

Inhibitors of BRAF^V600E^ kinase are currently under investigations in preclinical and clinical studies involving BRAF^V600E^ glioma. Studies demonstrated clinical response to such individualized therapy in the majority of patients whereas in some patients tumors continue to grow despite treatment. To study resistance mechanisms, which include feedback activation of mitogen-activated protein kinase (MAPK) signaling in melanoma, we developed a luciferase-modified cell line (2341^luc^) from a Braf^V600E^ mutant and Cdkn2a- deficient murine high-grade glioma, and analyzed its molecular responses to BRAF^V600E^- and MAPK kinase (MEK)-targeted inhibition. Immunocompetent, syngeneic FVB/N mice with intracranial grafts of 2341^luc^ were tested for effects of BRAF^V600E^ and MEK inhibitor treatments, with bioluminescence imaging up to 14-days after start of treatment and survival analysis as primary indicators of inhibitor activity. Intracranial injected tumor cells consistently generated high-grade glioma-like tumors in syngeneic mice. Intraperitoneal daily delivery of BRAF^V600E^ inhibitor dabrafenib only transiently suppressed MAPK signaling, and rather increased Akt signaling and failed to extend survival for mice with intracranial 2341^luc^ tumor. MEK inhibitor trametinib delivered by oral gavage daily suppressed MAPK pathway more effectively and had a more durable anti-growth effect than dabrafenib as well as a significant survival benefit. Compared with either agent alone, combined BRAF^V600E^ and MEK inhibitor treatment was more effective in reducing tumor growth and extending animal subject survival, as corresponding to sustained MAPK pathway inhibition. Results derived from the 2341^luc^ engraftment model application have clinical implications for the management of BRAF^V600E^ glioma.

## INTRODUCTION

Elevated mitogen-activated protein kinase (MAPK) signaling is common in adult and pediatric gliomas. Among the gene alterations that promote increased MAPK signaling is a single nucleotide substitution in BRAF kinase exon 15, which results in expression of the constitutively-active BRAF^V600E^ mutant kinase [1]. For pediatric glioma, mutation screening studies have revealed that BRAF^V600E^ frequencies are highest in grade II and III pleomorphic xanthoastrocytoma (PXA) (60-66%) [2]. BRAF^V600E^ has also been detected in 18-50% of ganglioglioma [3, 4], 5-33% of grade II-IV malignant astrocytoma [4–7], and 9% of pilocytic astrocytoma [4, 5]. We previously reported that five out of seven (71%) of BRAF^V600E^ mutant pediatric grade II-IV astrocytoma have homozygous deletion of CDKN2A, which encodes the p16 tumor suppressor [6]. A subsequent study reported that the BRAF^V600E^ mutation and CDKN2A deletion are more frequent in pediatric low-grade glioma that transform to high-grade malignancy than in non-transforming tumors [8]. BRAF^V600E^ is less common in adult glioma, in which MAPK signaling is most often stimulated by gene amplification and mutation of upstream receptor tyrosine kinases [9, 10]. Cumulatively, the results of mutation screening studies indicate that BRAF^V600E^ is important to multiple pediatric glioma types, and suggest that this oncogenic alteration cooperates with CDKN2A deletion to promote neoplastic transformation and tumor malignant progression.

In a previous study, we modeled the combined effects of BRAF^V600E^ expression and CDKN2A deficiency by crossing the cre-conditional *Braf^CA^* [11] and *hGFAP-cre* [12] mice to mice lacking *Ink4a-Arf* [13], a locus that contains the murine homolog of CDKN2A. Triple transgenic mice expressed Braf^V600E^ in Gfap+ cells under control of the endogenous Braf promoter, and lacked Cdkn2a expression [14]. These mice died prior to developing tumors but cells isolated from the ganglionic eminence of *Braf^CA^ Ink4a-Arf knock-out mice* and infected with adenovirus expressing cre recombinase (Ad-cre) in culture, became tumorigenic upon intracranial injection into SCID mice. We also observed intracranial tumor formation by inducing Braf^V600E^ expression and Cdkn2a deficiency through injection of Ad-cre into the subventricular zone (SVZ) of the lateral ventricle of *Braf^CA^* mice bred with a cre-conditional knock-out allele of *Ink4a-Arf (Ink4a-Arf^LoxP/LoxP (fl/fl)^)* [14].

Results from the use of Braf^V600E^ *Ink4a-Arf* knock-out murine allografts and BRAF^V600E^ + CDKN2A-deficient human glioma xenografts demonstrated the anti-tumor activity of PLX4720 [14, 15], a tool compound of the FDA-approved BRAF^V600E^-inhibitor vemurafenib. These studies helped motivate an active clinical trial for assessing vemurafenib in treating children with recurrent BRAF^V600E^ glioma (ClinicalTrials.gov Identifier NCT01748149). There are early indications that this personalized approach benefits some patients with BRAF^V600E^ positive ganglioglioma [16, 17], recurrent PXA [18] and recurrent glioblastoma [19]. Moreover, patients with relapsed or refractory high-grade and low-grade BRAF^V600E^ glioma have shown radiographic response to treatment with BRAF^V600E^ inhibitor dabrafenib in a phase 1 clinical trial. In some cases, however, tumors showed progression despite dabrafenib treatment, suggesting that some glioma have inherent, primary resistance to BRAF^V600E^ targeted therapy [20]. The observation of progressive tumor growth during treatment is consistent with our more recent preclinical studies that showed no significant impact on survival rates from PLX4720 monotherapy when treating mice with distinct BRAF^V600E^ mutant and CDKN2A deficient tumors models (intracranial xenografts from pilocytic astrocytoma [21] and glioblastoma [22]).

Here, we present results from the characterization and therapeutic testing of a newly developed Braf^V600E^-expressing Cdkn2a deficient glioma model, the first to involve the use of Braf^V600E^ glioma cells in a syngeneic, immunocompetent host. Our study examines the relative anti-tumor activity of BRAF^V600E^ vs. MEK targeted monotherapy, and of combination therapy using the same inhibitors. Compared with the effects of either inhibitor alone, combination therapy significantly decreased Ki67 positivity, reduced bioluminescence signaling, and conferred the most substantial survival benefit to animal subjects with lentivirus-luciferase modified, Braf^V600E^ expressing *Ink4a-Arf* knock-out murine allografts. Our results demonstrate the utility of this model for testing small molecule inhibitors, and should as well, prove useful for testing therapies for modulating immune response against BRAF^V600E^ mutant glioma.

## RESULTS

### Braf^V600E^ + Ink4a-Arf deficient 2341^luc^ cells produce intracranial tumors in FVB/N mice with features characteristic of high-grade glioma

**Figure 1:**
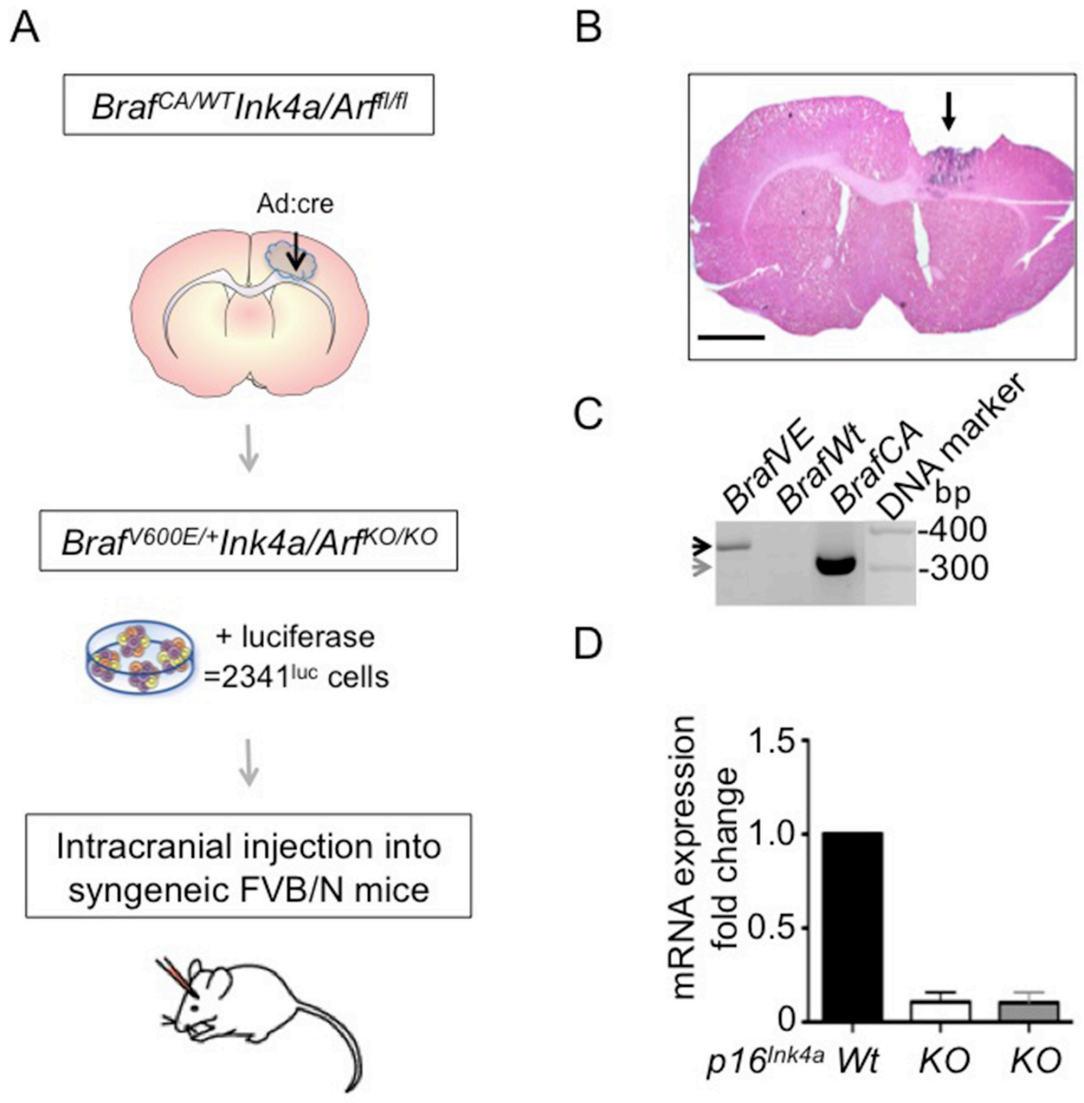
Braf^V600E^ mutant Ink4a-Arf deleted tumor-derived cells form syngeneic grafts **A.** Schematic of 2341^luc^ model development. Cells were isolated from a tumor developed in an 18-week-old *Braf^CA/+^*Ink4a/Arf*^LoxP/LoxP (fl/fl)^* mouse (animal number 2341) that had received adenovirus-cre (Ad:cre) virus injection in the corpus callosum at ten weeks of age. Tumor cells were subsequently modified with lentivirus expressing luciferase (2341^luc^), for injection into syngeneic FVB/N mice. **B.** Hematoxylin and Eosin staining of a tumor developed by injection of Ad:cre as described in A. Arrow points to the location of the tumor and parts of the tumor were excised for cell culturing. Scale bar is 1000 μm. **C.** PCR detection of mutant *Braf^V600E^* and *Braf^CA^* alleles. Specific primers were used to distinguish between the 308 bp band for cre-conditional *Braf^CA^* (grey arrow) and the 335 bp band for *Braf^V600E^* (black arrow). Panel depicts 2341^luc^ cells, which express *Braf^V600E^* (*BrafVE*), control subventricular zone-derived cells with the wild type *Braf* (*BrafWt*) or the *Braf^CA^* (*BrafCA*) allele. **D.** QPCR detection of *p16^Ink4a^*. Reduced expression of *p16^Ink4a^* mRNA was detected by real-time PCR in 2341^luc^ cells (KO; white bar), and subventricular zone-derived neurosphere cells homozygous deleted for *Ink4a-Arf* (KO; grey bar). The mRNA expression levels are normalized to expression levels in subventricular zone-derived wild type (Wt) cells, used as control.

To establish a tumor-derived glioma cell line carrying the Braf^V600E^ mutation and deficient for Cdkn2a, we injected adenovirus expressing cre recombinase (Ad-cre) into the corpus callosum of ten week-old, cre-conditional, FVB/N *Braf^CA/+^*Ink4a/Arf*^LoxP/LoxP (fl/fl)^*transgenic mice (Figure 1A). Mice consistently developed tumors by eight weeks post-injection (Figure 1B). Tumor isolated from one such animal (animal number 2341) was used in preparing an explant culture that was subsequently expanded and modified with firefly luciferase-encoding lentivirus (2341^luc^; Figure 1A). PCR analysis of DNA extracted from these cells showed that the *Braf^V600E^* transgene was expressed (Figure 1C). Deletion of *Ink4a-Arf*, which encodes *p16^Ink^*^4a^ and *p19^Arf^*, was confirmed by quantitative PCR for *p16^Ink^*^4a^ (Figure 1D).

To determine whether the 2341 cell line has acquired additional genetic changes in selected oncogenes, and in particular during modification with luciferase lentivirus, we analyzed 2341 cells at initial passage, and following lentiviral modification and expansion. Targeted sequencing of known oncogenic driver genes, including *Akt3, Kras, Map2K1, Pik3ca, Pik3r2, Pten, H3f3a* and *H1h3a* showed no alteration from wild-type sequence for these genes. This result supports earlier observations that expression of Braf^V600E^ and deletion of Cdkn2a are sufficient for neoplastic transformation (Supplementary Figure S1) [14].

**Figure 2:**
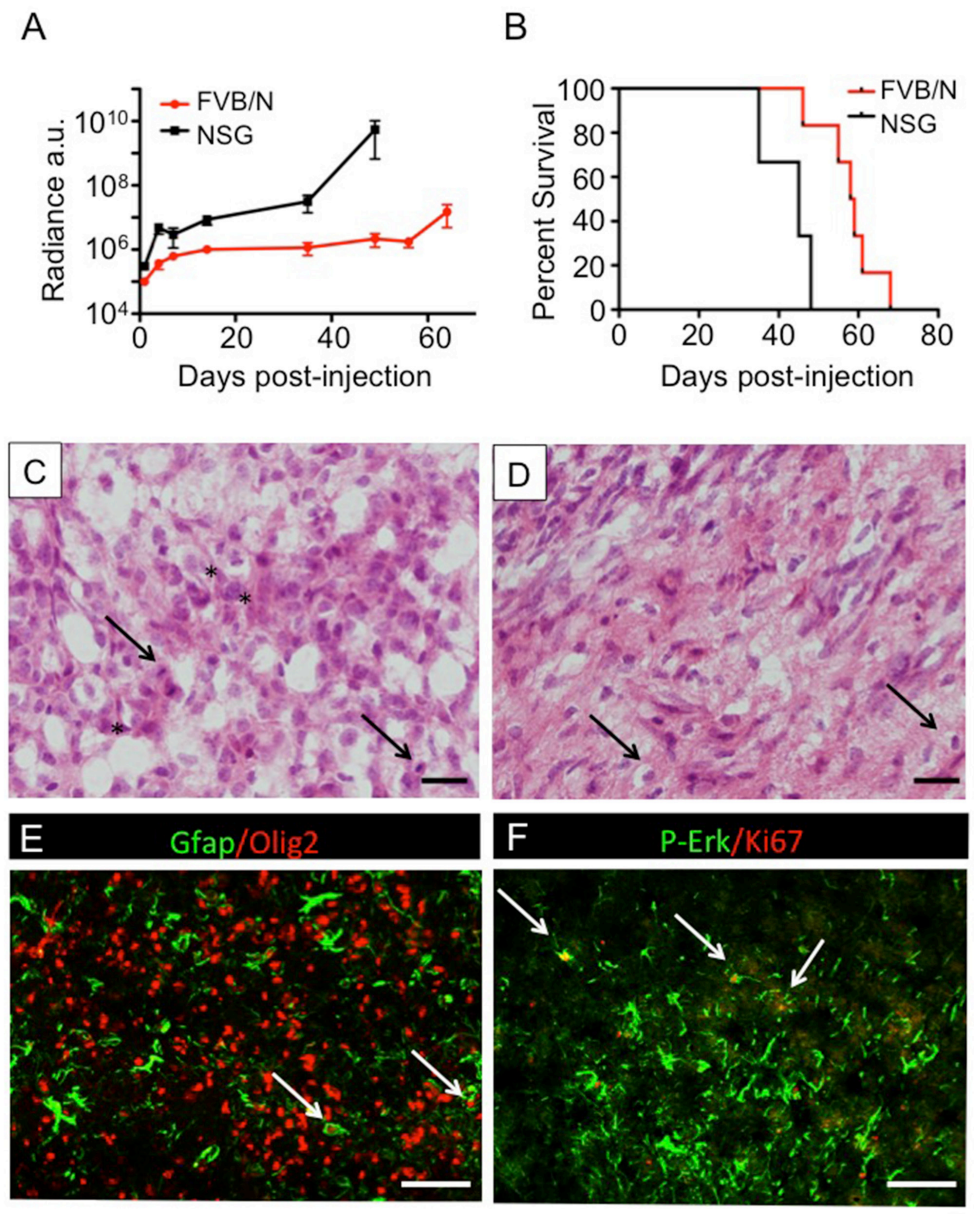
2341^luc^ syngeneic grafts showed characteristics of high-grade glioma **A.** Bioluminescence imaging (BLI) growth plots of intracranial tumors established from 2341^luc^ cells in syngeneic, immunecompetent FVB/N and immunecompromised Nod scid gamma (NSG) mice, respectively. Values are displayed in arbitrary units (a.u.). The linear plots of log base 10 values indicated that exponential increase of bioluminescence was achieved by all tumors, though the rate of increase was slower in FVB/N mice. Standard deviations are indicated. **B.** Kaplan-Meier survival plots for 12 NSG and 10 FVB/N mice, respectively, following intracranial injection of 30,000 2341^luc^ cells per animal. The median survival post-injection is 58.5 days for FVB/N mice and 45 days for NSG mice (*=p=0.010). **C, D.** H&E-stained tumor tissues showed histopathological features of high-grade glioma, including mitotic figures (C; arrows), pleomorphic and hyperchromatic nuclei (C; asterisks) and invading tumor cells (D; arrows). **E, F.** Immunofluorescence of 2341^luc^ syngeneic grafts for astrocyte marker Gfap (green), oligodendrocyte lineage and glioma cell marker Olig2 (red) (E) phospho-Erk (p-Erk; green) and proliferation antigen Ki67 (red), is indicative of a high-grade astrocytic tumor (F) White arrows point to double-positive cells. Scale bars are 20 μm in C, D and 200 μm in E, F.

Serial bioluminescence imaging (BLI) of 2341^luc^ cells, following intracranial injection of 30,000 cells in immunodeficient, Nod scid gamma (NSG) mice, as well as in immune competent, syngeneic FVB/N mice, revealed progressive increase in luminescence in all injected mice. The increase in luminescence is more rapid in the NSG animals compared with the FVB/N animals (Figure 2A). Median symptom-free survival of mice for NSG and FVB/N mice was 45 and 58.5 days, respectively (p=0.01; Figure 2B).

H&E staining of sections from resected brains showed tumor that was highly cellular, infiltrative, and pleomorphic (Figures 2C and 2D). Tumors expressed the astrocyte marker Gfap, oligodendrocyte lineage and glioma marker Olig2, proliferative marker Ki67, and phosphorylated extracellular kinase (phospho-Erk), indicative of active MAPK pathway signaling (Figures 2E and 2F).

### Braf^V600E^-expressing Ink4a-Arf knock-out 2341^luc^ tumor cells become unresponsive to BRAF^V600E^-targeted inhibition

**Figure 3:**
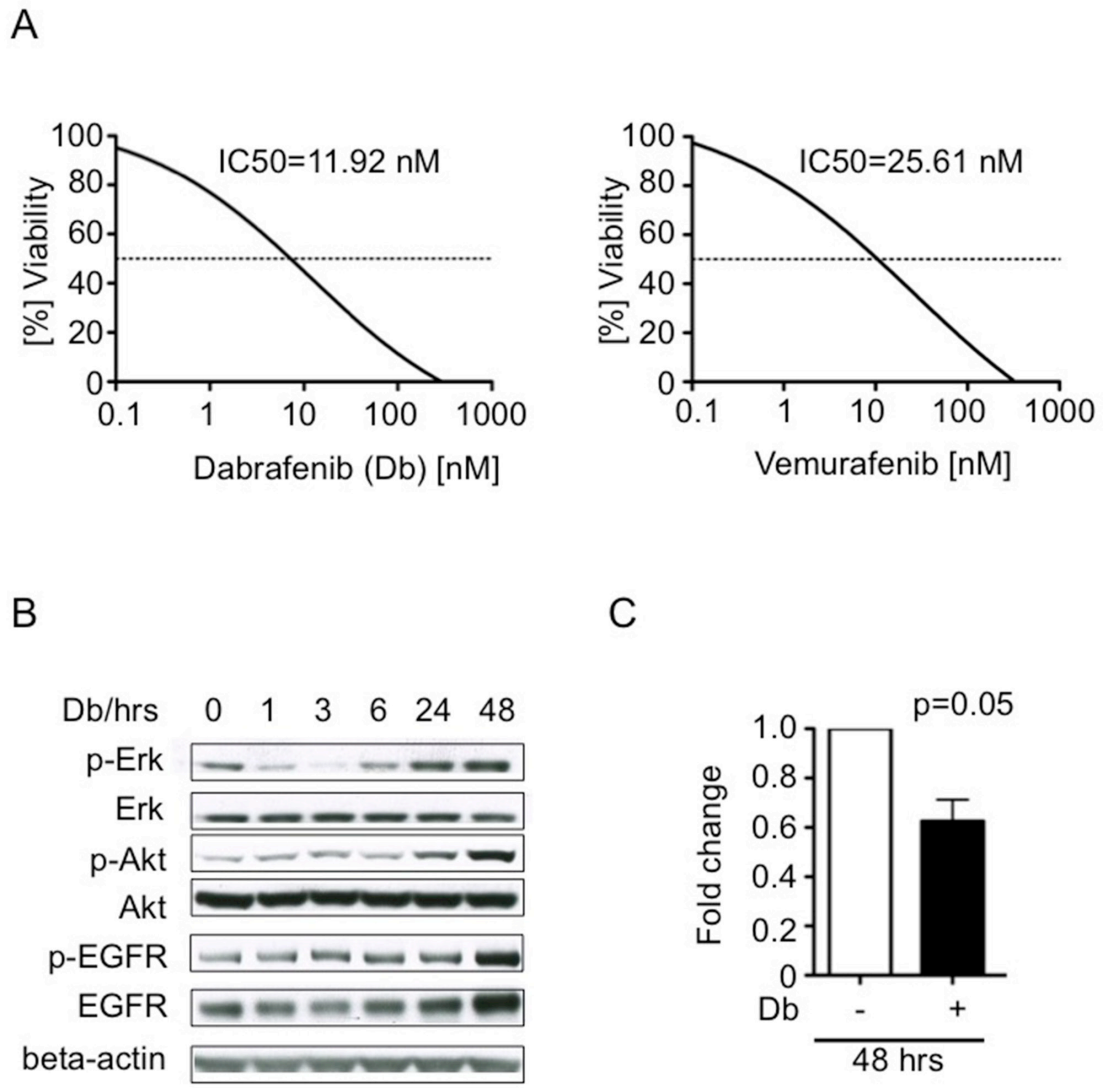
Tumor cell response to BRAF^V600E^ inhibitors dabrafenib and vemurafenib *in vitro* **A.** Dose-response-curves showing *in vitro* viability effects of increasing concentrations of dabrafenib (Db; left panel) and vemurafenib (right panel) on 2341^luc^ cells (n= 3). **B.** EGFR, Akt and Erk activities increase in 2341^luc^ cells during treatment with BRAF^V600E^ inhibitor Db. Cells were treated with Db at IC50 concentration for up to 48 hours (hrs) and protein was extracted following a 20 min stimulation with EGF (25 ng/ml), and analyzed by immunoblotting for phospho-Erk (p-Erk), total Erk (Erk), phospho-Akt (p-Akt), total Akt (Akt), phospho-EGFR (p-EGFR) and total EGFR (EGFR). After an initial decrease, p-ERK signal increased from six to 48 hrs, p-Akt signal increased from 24 to 48 hrs and p-EGFR as well as EGFR increased at 48 hrs, beyond phosphorylation levels detected before treatment was started. Beta-actin was detected as loading control. **C.** Short-term Db treatment inhibits 2341^luc^ cell proliferation. Cells were treated with IC50 concentrations of Db for 48 hrs, incubated with Hoechst dye, and analyzed for DNA content (HO>2), indicative of actively proliferating cells, by flow cytometry. The graph shows fold change in proliferative cells (p=0.05).

We compared the effects of treating 2341^luc^ cells, *in vitro*, with vemurafenib, which continues to see extensive use in treating BRAF^V600E^ mutant cancer, as well as with a second FDA-approved inhibitor of BRAF^V600E^, dabrafenib (Db; GSK2118436). Results from inhibitor treatment indicated IC50 values of 11.92 nM for Db and 25.61 nM for vemurafenib (Figure 3A). A human BRAF wild-type pediatric glioma cell line (SF188) exhibited no significant response to Db up to concentrations as high as 0.5 μM. Vemurafenib concentrations as high as 10 μM only reduced cell viability by 40% (Supplementary Figure S2). The latter result suggests off-target effects of vemurafenib that have been reported by others [27].

We further investigated the 2341^luc^ cell response to BRAF^V600E^ inhibitor Db by examining phospho-Erk (p-Erk) levels, a commonly used surrogate of MAPK pathway activity. Immunoblot results showed p-Erk levels decreasing over the first six hours of exposure to dabrafenib, with increases evident beyond 24 hours, and that ultimately exceeded the phosphorylation levels of untreated cells (Figure 3B). Based on our previous work [21] as well as the published work of others [28–31] (Supplementary Figure S3), we analyzed EGFR and PI3k-Akt activity in Db-treated 2341^luc^ cells, as potential mediators of MAPK signaling rebound. Immunoblot results showed increased phospho-Akt (p-Akt) from 24 to 48 hours, and increased phospho-EGFR (p-EGFR) as well as total EGFR at 48 hours (Figure 3B).

Db treatment was next examined for anti-proliferative activity. For this, cells were treated for 48 hours at Db IC50 concentrations, and then analyzed for DNA content by flow cytometry. Db treated cell samples showed significantly reduced DNA content relative to untreated cells, indicative of reduced proliferation (Figure 3C).

### Combination therapy is more effective than trametinib or dabrafenib monotherapy in preventing MAPK activation and inhibiting tumor cell proliferation

**Figure 4:**
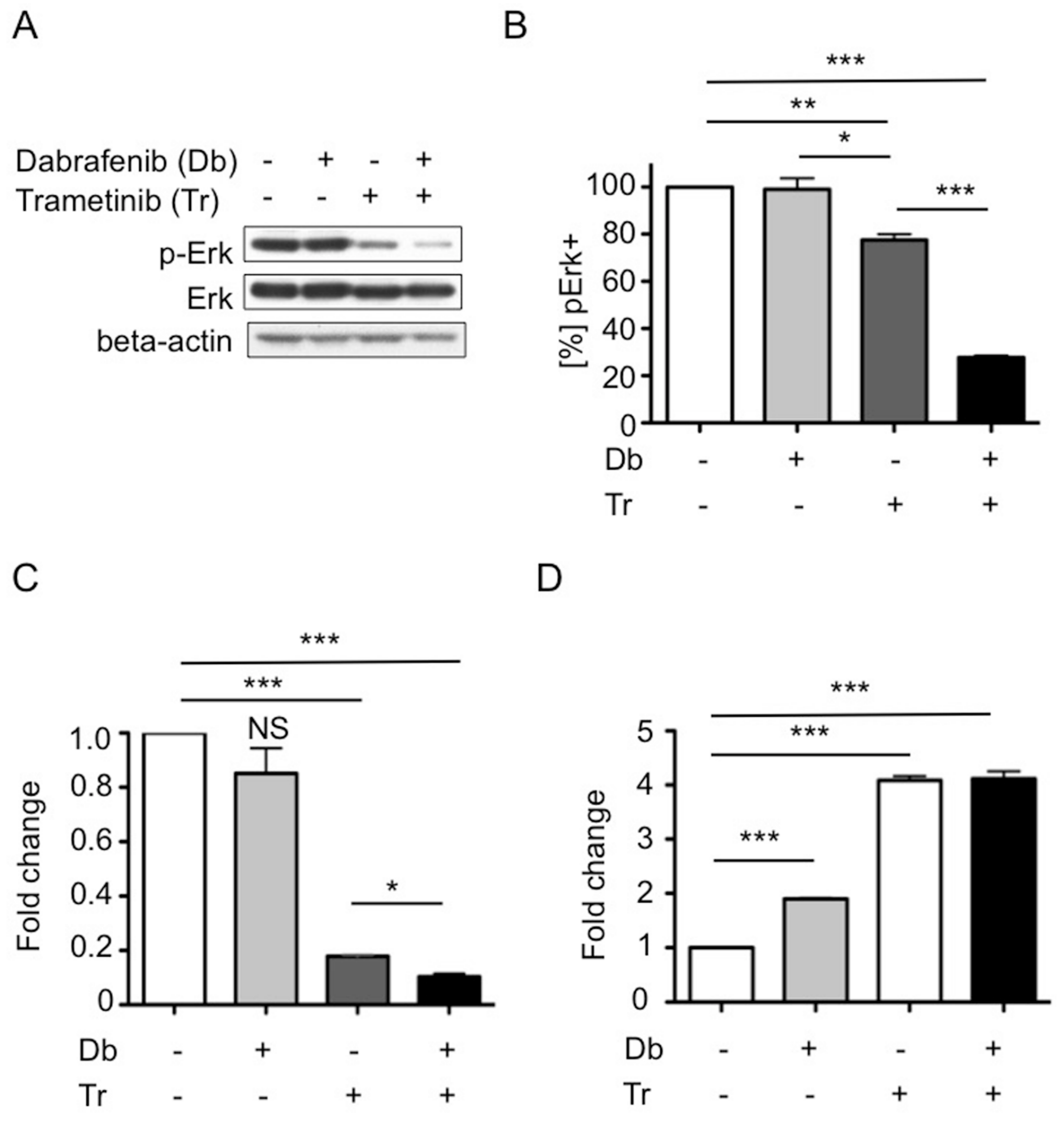
Combined BRAF^V600E^ and MEK inhibition effects on MAPK pathway activity, tumor cell growth and apoptosis **A, B.** Combined BRAF^V600E^ and MEK inhibition inhibits p-Erk levels more effectivey than MEK inhibition alone. (A) 2341^luc^ cells treated with IC50 doses of dabrafenib (Db), trametinib (Tr) or a combination of both (Db+Tr) for four days and stimulated with EGF (25 ng/ml) for 20 min before protein extraction were analyzed by immunoblotting for p-Erk and total Erk (Erk). Beta-actin was detected as loading control. P-ERK was suppressed by Tr and further suppressed by combination treatment. (B) Flow cytometric quantification of p-Erk levels in 2341^luc^ cells treated with Db, Tr or a combination of both (Db+Tr) for five days. Db did not alter p-Erk levels. Tr significantly decreased p-Erk-positive cell frequency (**=p=0.0060 for control versus Tr) and combined Db and Tr treatment resulting in further decrease (***=p<0.0001 for Tr versus Db + Tr and for control versus Db + Tr; *=p=0.0412 for Db versus Tr). Graph shows percent p-ERK-positive ([%] pERK+) cells normalized to control-treated cells = 100%. **C.** Combination treatment reduces 2341^luc^ cell cycling more effectively than single agent treatment. Cells were treated with IC50 concentrations of Db, Tr or a combination of both (Db+Tr) for four days, incubated with Hoechst dye, and analyzed for DNA content (HO>2) by flow cytometry. Graph shows fold change in the frequency of cells with a DNA content of HO>2. Tr but not Db decreased proliferation significantly (***=p=0.0001 for Tr versus control) and this effect was enhanced by combination treatment (***=p<0.0001 for Db+Tr versus control; *=p=0.0149 for Db+Tr versus Tr) (n=3). Graph depicts the fold change of frequency of proliferative cells normalized to control-treated cells = 1. **D.** Single agent and combination treatment increases apoptosis. Cells were treated as in **C.** and frequency of annexin V-positive cells was determined as surrogate for apoptotic cells. Single drug Db and Tr treatment and, more effectively, a combination of both significantly increased apoptosis (***=p<0.0001 for Db versus control and Tr versus control; ***=p=0.0002 for Db+Tr versus control). Graph depicts fold change of annexin V-positive cell frequency normalized to control-treated cells = 1.

Clinical results using Db in combination with the MEK inhibitor trametinib (Tr) to treat melanoma showed improved progression-free survival, compared to either inhibitor used as monotherapy [32]. To evaluate the potential of this combination therapy for treating BRAF^V600E^ high-grade glioma, we first compared single vs. dual agent effects on 2341^luc^ cell cultures. After determining the IC50 value for Tr (IC50 = 30.5 nM) in 2341^luc^ cells (Supplementary Figure S4), we found this concentration to be effective at decreasing p-Erk levels in EGF stimulated cells (25 ng/ml). This suppressive effect was further augmented by combining Tr with Db (Figure 4A). Flow cytometry for p-Erk using 2341^luc^ cells, treated for five days with IC50 concentrations of both agents, showed that Tr but not Db significantly decreased p-Erk-positive cell frequency, with combination treatment resulting in further decrease (p<0.001 for Tr versus Db + Tr; Figure 4B).

Single and dual agent treatments were also examined for anti-proliferative and pro-apoptotic activities. Cells treated for 4 days with IC50 concentrations of Db, Tr or a combination, were analyzed by flow cytometry for DNA content of HO>2, which is indicative for proliferative cells. Db had no significant effect on proliferation. In contrast, Tr significantly decreased proliferation (p=0.0001 for Tr versus control) and, as above, this effect was enhanced by adding Db (p=0.0149 for Db + Tr versus Tr; Figure 4C). In parallel, cells were analyzed for apoptosis by Annexin V staining, with results showing that all treatments significantly increased apoptotic cell fractions, with the most substantial increases observed for Tr only and combination treatments (Figure 4D).

### Combination therapy effectively prevents MAPK activation, proliferation and increases apoptosis in orthotopic tumors

**Figure 5:**
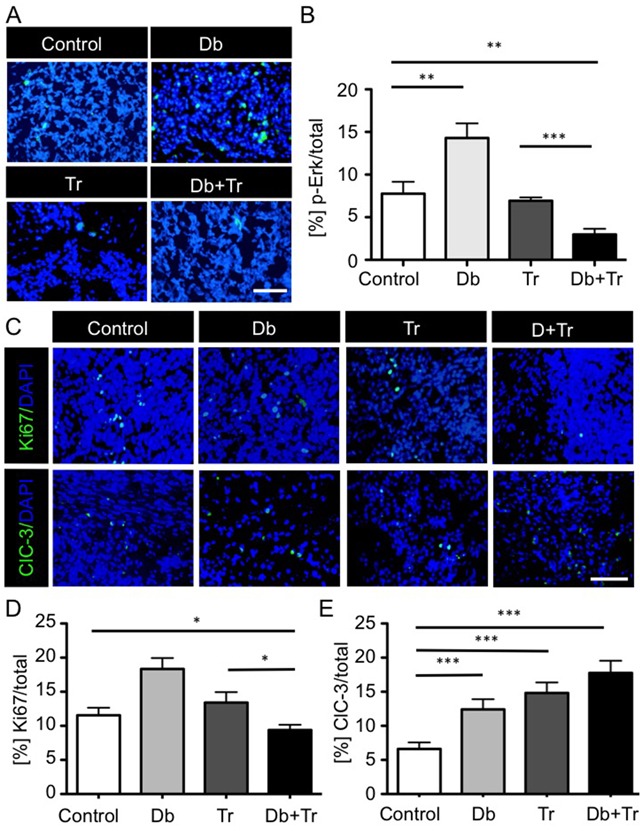
BRAF^V600E^ and MEK inhibitor mono- and combination therapy effects on MAPK pathway activity, proliferation and apoptosis in syngeneic 2341^luc^ grafts **A, B**. Combined BRAF^V600E^ and MEK inhibition inhibits p-Erk levels more effectively than MEK inhibition alone. A. Immunofluorescence for phospho-Erk (p-Erk) in 2341^luc^ syngeneic grafts treated with dimethylsulfoxide (Control), dabrafenib (Db), trametinib (Tr), or a combination of both (Db+Tr), for 14 days starting at day 30 post-implantation (n=8/group). P-Erk staining is in green. DNA is stained with DAPI and is in blue. Scale bars are 20 μm. **B.** Quantification of p-Erk+ tumor cells in 2341^luc^ syngeneic grafts for each treatment group. P-Erk+ cell frequency was determined using Image J. Db monotherapy increased p-Erk+ cell frequency (**=p=0.0075 for Db versus control). Tr monotherapy decreased pErk+ cell frequency, compared with Db monotherapy (**p<0.0023 for Tr versus Db), but not with control (p=0.31). Tr in combination with Db significantly decreased p-Erk + cell frequency (**=p=0.004 for Db+Tr versus control; ***=p=0.0007 for Db+Tr versus Tr; ***=p<0.0001 for Db+Tr versus Db). **C–E.** Effects of 14-days consecutive inhibitor treatment on proliferation and apoptosis. **C.** Immunofluorescence images of Ki67 (top panels) and cleaved caspase-3 (ClC-3; bottom panels) stained tumor tissue from 2341^luc^ syngeneic grafts at day 14 of treatment (n=8/group). Ki67 and ClC-3 are in green. DNA was stained with DAPI and is shown in blue. Scale bar is 20 μm. **D, E.** Quantification of Ki67+ and ClC-3+ cells in tumors from each treatment group. **D.** Db or Tr monotherapy did not significantly change Ki67+ Db or Tr monotherapy did not significantly change Ki67 positivity. Combination treatment significantly decreased Ki67+ cell frequency (*=p=0.0339 for Db+Tr versus control; *=p=0.017 for Db+Tr versus Tr). **E.** Each treatment significantly increased apoptosis compared with control (***=p=0.0008 for Db versus control; ***=p<0.0001 for Db+Tr versus control; ***=p<0.0001 for Db+Tr versus Tr).

We next examined the effects of treating FVB/N mice, bearing syngeneic, orthotopic 2341^luc^ tumors, using Db and Tr mono- and combination therapies. Brains with tumor, resected at the end of 14-days treatment, were stained for p-Erk (Figures 5A and 5B). Single agent Db increased p-Erk-positive (p-Erk+) tumor cell frequency (p=0.075), while Tr monotherapy had insignificant effect, relative to control (p=0.31). Consistent with the *in vitro* data, Tr in combination with Db significantly decreased p-Erk+ cell frequency (p=0.004 for Db+Tr versus control). The combination effect was significant in comparison with each monotherapy as well (p=0.0007 for Db+Tr versus Tr; p<0.0001 for Db+Tr versus Db; Figure 5B). We compared differences in p-Akt between treated groups at two timepoints during treatment. We observed an increase of p-Akt levels after three days of Db treatment (Supplementary Figure S5), consistent with the increase within 48 hrs of Db treatment *in vitro* (Figure 3B). P-Akt levels increased further within 14 days of Db treatment, in association with adaptive resistance. Combined Db and Tr therapy prevented the increase of p-Akt levels (Supplementary Figures 5A and 5B).

We also examined treatment effects on tumor cell proliferation and apoptosis by staining for Ki67 and cleaved caspase-3, respectively. Whereas Db monotherapy significantly increased Ki67 positivity and Tr monotherapy showed no significant effect in relation to control, combination therapy significantly decreased Ki67 positive cells (p=0.0339 for Db+Tr versus control; p=0.017 for Db+Tr versus Tr; Figures 5C and 5D). As treatment effects on apoptosis were concerned, all therapies significantly increased cleaved caspase-3 positivity, with the most substantial increase observed for combined inhibitor treatment (Figures 5C and 5E).

### Combination therapy effectively inhibits tumor cell growth and extends survival

**Figure 6:**
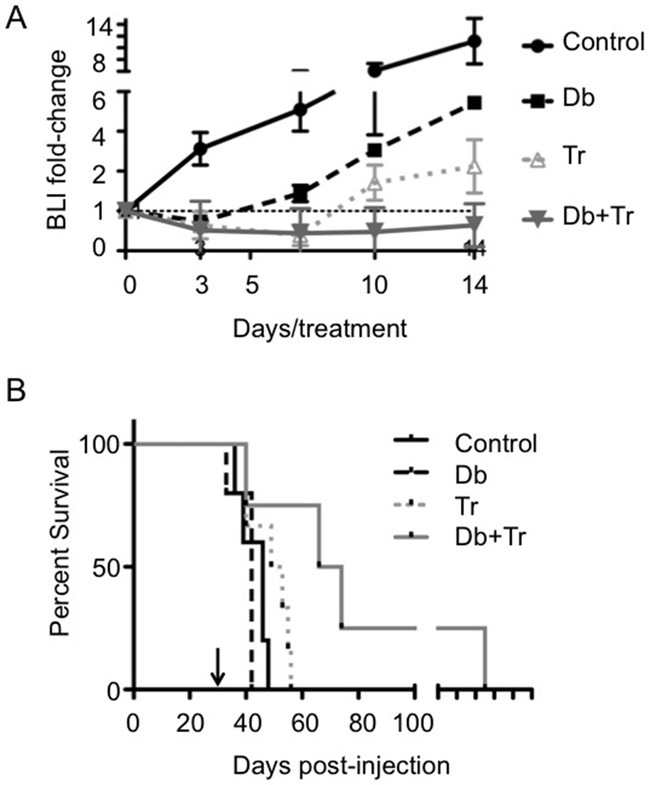
BRAF^V600E^ and MEK inhibitor mono- and combination therapy effects on bioluminescence intensity and survival in syngeneic Braf^V600E^ expressing *Ink4a-Arf* knock-out grafts **A.** Dabrafenib (Db) and trametinib (Tr) combination therapy but not monotherapy shows durable reduction of bioluminescence intensity (BLI). Plots for BLI of 2341^luc^ syngeneic grafts over 14 day treatments with Db, Tr, or a combination of both (Db+Tr) (n=8/group). Treatment was initiated at day 30 post-implantation of 2341^luc^ and BLI was measured on treatment days 3, 7, 10, and 14, and expressed as normalized BLI (fold-change from the start of treatment). **B.** Db and Tr combination therapy but not monotherapy significantly extends survival. Graph shows Kaplan-Meier survival plot of mice with 2341^luc^ syngeneic grafts treated with Db or Tr monotherapy or a combination of both, until they developed neurologic symptoms. Db monotherapy (median survival = 42 days) failed to extend survival while Tr monotherapy significantly increased survival (median survival Tr = 51 days; median survival control = 46 days; p=0.044 for Tr versus control). The combination treatment was most effective in extending survival (median survival = 70 days; p=0.049 for Db+Tr versus control).

Effects of treatment on lentivirus-luciferase modified, syngeneic, orthotopic 2341^luc^ tumors were assessed by serial bioluminescence imaging (BLI) of mice treated with mono- or combination therapy. The results showed that Db and Tr monotherapy reduced BLI for three and seven days, respectively, following treatment initiation. Beyond these time-points, BLI values increased, despite continuously administrating inhibitor daily. In contrast, combination therapy inhibited BLI over the entire period of treatment (14 days; Figure 6A). Consistent with the changes in BLI, Db monotherapy provided no survival benefit (median survival control = 46 days versus median survival Db = 42 days: p = 0.253). Tr monotherapy increased survival significantly in relation to control group mice (median survival= 51 days; p=0.044 for Tr versus control), and combination therapy further extended survival (median survival = 70 days; p=0.049 for Db + Tr versus control; Figure 6B).

## DISCUSSION

The BRAF^T1799A^ point mutation, which results in the expression of constitutively active BRAF^V600E^ kinase, occurs in many cancers, including gliomas. Array CGH and exome sequencing of a population of matched pediatric low-grade glioma (PLGG) and secondary high-grade glioma (sHGG) identified *BRAF^V600E^* mutation and CDKN2A deletion as the most recurrent alterations in sHGG, at 39% and 57% respectively [8]. This study determined that *BRAF^V600E^* identifies a clinically distinct subset of patients whose tumors are more likely to undergo malignant progression [8].

Earlier, we assessed whether BRAF activation alone would be sufficient for tumor formation, by inducing expression of Braf^V600E^ under control of its endogenous promoter in Gfap+ cells, by breeding Braf^CA/+^ hemizygous and hGFAP-cre transgenic mice [14]. We found that mice only survived for up to three weeks of age without any evidence of tumor formation. We concluded that Braf^V600E^ expression during development is associated with lethality unrelated to glioma formation, and that Braf^V600E^ expression alone is insufficient to induce gliomagenesis during the first month of age. To test whether Braf^V600E^ cooperates with Cdkn2a (*Ink4a-Arf*) deletion to induce glioma formation, we injected Ad-cre near the SVZ of adult *Braf*^*CA/+*^ and *Braf*^*CA/+*^ *Ink4a-arf*^*fl/fl*^ mice, respectively. Combined expression of Braf^V600E^ and homozygous deletion of *Ink4a-Arf*, but not expression of Braf^V600E^ alone, induced gliomagenesis. These data corroborated that Braf^V600E^ expression cooperates with homozygous inactivation of Cdkn2a to generate tumors [14]. In the current study, we obtained tumors by injecting Ad-cre into the corpus callosum of *Braf*^*CA/+*^ *Ink4a-arf*^*fl/fl*^ mice, which were genetically identical to the mice used in the earlier study (Figures 1A and 1B). From one such tumor (#2341), we have developed a syngeneic and orthotopic Braf^V600E^ mutant and Cdkn2a deficient engraftment model. Our current findings shows that non-SVZ cells expressing the Braf^V600E^ mutant and lacking Cdkn2a are capable to give rise to transplantable tumors.

Established human Braf^V600E^ GBM lines are capable of generating xenografts and are typically kept in serum-conditions. Prolonged maintenance of cell cultures in the presence of serum significantly alters transcriptional and genomic cell profiles, in comparison with their parental tumor. In contrast, serum-free conditions, such as the ones used to maintain the 2341 cells, preserve better the expression profiles of the parental tumors [33, 34]. The 2341^luc^ experimental glioma model thus provides a tool complementary to the already existing human BRAF^V600E^ cell lines.

Histopathology and immunohistochemical analyses of 2341^luc^ syngeneic grafts identified high-grade astrocytoma-like features and tumor cell phosphorylation of Erk, a surrogate for MAPK pathway activation. Tumors also co-expressed astrocyte and neural stem cell marker Gfap and Olig2, which is rare in normal brain cells, and indicates aberrant cell fate specification typical of tumor cells (Figures 2C and 2F) [35].

We found that vemurafenib and Db had IC50 values in 2341^luc^ cells in the nanomolar range (Figure 4A), which is consistent with IC50 values of Db in some human melanoma cells [36]. With Db being more specific for Braf^V600E^ than vemurafenib in 2341^luc^ cells (Supplementary Figure S2) and its use in clinical trials for BRAF^V600E^ mutant glioma [20], we focused subsequent analyses on effects of Db on 2341 cells and grafts. In total, our data support transient 2341 cell response to Db, as indicated by effects on MAPK pathway activity and cell proliferation (Figures 4B and 4C). MAPK pathway activity is restored, and in addition Akt and EGFR activities are heightened beyond six hours of Db treatment, as indicated by phosphoprotein immunoblot analyses of Db-treated cells (Figure 3B). Moreover, mice carrying 2341 grafts showed elevated p-Akt levels after three days of Db treatment, when compared with control and combination treatment (Supplementary Figure S5). The transient effect of Db on p-Erk levels and subsequent increase in p-Akt suggests the ability of 2341 cells to adapt to BRAF^V600E^ inhibition in a manner similar to that seen in melanoma, where mutant BRAF inhibition provides strong selective pressure for upregulation of MAPK and PI3k-Pten-Akt pathways through epigenetic and genetic mechanisms [37].

Moreover, primary resistance or adaptive tumor response to BRAF inhibition in melanoma include upregulation of receptor tyrosine kinases [30]. A point of distinction between glioma and melanoma adaptation to BRAF^V600E^ inhibition involves EGFR. We have previously shown that human BRAF^V600E^ glioma but not melanoma cell lines express high levels of EGFR. Moreover, BRAF^V600E^ inhibition of glioma cells with the vemurafenib tool compound PLX4720 releases a negative feedback loop that is placed on EGFR signaling by BRAF^V600E^ [21]. This relationship has also been observed in BRAF^V600E^ colorectal cancer [38] and the increase in pEGFR as well as total EGFR levels in Db-treated 2341 cells (Figure 3B), suggests a similar feedback mechanism in these mouse glioma cells (Supplementary Figure S3). Emerging clinical data and our preclinical study argue for a similar mechanism in glioma where BRAF mutation does not always appear to be predictive for responsiveness to BRAF inhibitor therapy.

The occurrence of acquired resistance to BRAF^V600E^ inhibition in the treatment of various types of cancer [39–42] has motivated efforts to develop combination therapies to prevent and/or delay tumor adaptation. BRAF^V600E^ + MEK inhibitor combination treatment of melanoma patients with BRAF^V600E^ mutant tumors have shown this to be an effective strategy, as indicated by the survival benefit to this patient population [32]. These studies have motivated FDA-approval of Tr, a potent mitogen-activated protein kinase-1 (MEK)-1/2 inhibitor, for use in combination with Db as first-line therapy for metastatic melanoma. A further reason to combine BRAF and MEK inhibitors in patients is to decrease the development of secondary RAS-driven cancers, such as squamous cell carcinomas [43] and chronic lymphoblastic leukemia [44], that have been reported in patients inhibitor treated with BRAF monotherapy. These cancers develop due to the paradoxical activation of MAPK signaling in BRAF wild-type cells with upstream activation of RAS when treated with BRAF inhibitors. This MAPK activation is significantly impeded through the addition of MEK blockade [45].

Here, our results show that the BRAF^V600E^ inhibitor Db has modest effect on intracranial tumor growth (Figures 6A and 6B), with rapid and substantial increases in MAPK and Akt pathway activities (Figures 5A and 5B; Supplementary Figure S5). Using this regiment, Db reportedly showed remarkable efficacy in reducing tumor size in the brains of patients with brain metastasis [41] and an intact blood-brain-barrier only partially prevents Db from entering the brain [24].

Presumably, as a result of MAPK activity rebound and rapidly increasing Akt activity, however, Db monotherapy had virtually no effect on animal subject survival (Figure 6B). Tr decreased 2341 MAPK pathway activity and cell proliferation while increasing apoptosis *in vitro* (Figures 4A-4D), and this translated to a small but significant survival benefit for Tr-treated animals bearing orthotopic 2341 tumors (Figure 6B). Nonetheless, our results shown that 2341 grafts adapt to Tr monotherapy, whereas combination treatment delayed acquired resistance (Figure 6A). Our data are consistent with those obtained by studying human BRAF^V600E^ mutant glioma cell lines, which point to re-activation of MAPK pathway and increased AKT and EGFR signaling activity as a mechanisms of adaptation and resistance to BRAF^V600E^ inhibition (Supplementary Figure S3) [21, 46]. These similarities suggest that our mouse model has clinical relevance. Future studies will examine the effects of extended combination therapy, including the responses of cancer stem cell subpopulations.

The 2341^luc^ model exhibits multiple features of a useful preclinical engraftment model, as discussed in Oh et al [47], and include *in vivo* recapitulation of the diagnostic histopathology features of a human high-grade astrocytoma, *in vitro* tumor cell sustainability and amenability to genetic manipulation, engraftment consistency, and reproducible as well as predictable growth characteristics. As a result of 2341 cells being syngeneic to immunocompetent FVB/N mice, we anticipate our model as facilitating investigation of immunotherapy + small molecule inhibitor treatment of BRAF^V600E^ gliomas.

## MATERIALS AND METHODS

### Isolation of Braf^V600E^ Ink4a-Arf deleted tumor cells and cell culturing

Mouse breeding and procedures were conducted under an approved Animal Study Protocol according to UCSF Animal Care and Use Committee guidelines. Adenovirus-cre (Vector-BioLabs) was injected intracerebral using the following coordinates 1 mm anterior, 1 mm lateral and 2 mm deep to target the corpus callosum of *Braf^CA/+^*Ink4a/Arf*^LoxP/LoxP (fl/fl)^* young adult FVB/N mice to induce Braf^V600E^ expression under control of the endogenous Braf promoter [11], and deletion of p16^Ink4a^ and p19^Arf^. At eight weeks post-injection, we sacrificed mice because they exhibited neurologic symptoms and harvested tumor tissue and dissociated it with the Neural Dissociation Kit (Miltenyi Biotec Inc., San Diego) according to manufacturer's instructions. Primary cells were passaged using Accutase (Innovative Cell technologies) in non-adherent conditions at 37°C under 5% CO2 in Neurobasal-A media (1x B27-A, 1x N2, pen/strep, L-glutamine, 5 ng/ml EGF, 5 ng/ml bFGF2 and 5 ng/ml PDGF).

### Genotyping, PCR and targeted sequencing

Genomic PCR to distinguish between *Braf^CA^* and *Braf^V600E,^* was conducted as described [11]. Isolation of genomic DNA from cells was performed using 0.5 mg/ml Proteinase K for one hour followed by precipitation with 0.1 M sodium acetate and ethanol extraction. Primers were 5′-tgagtatttttgtggcaactgc (forward) and 5′-ctctgctgggaaagcggc (reverse) for *Braf*. Semi-quantitative PCR for *p16^Ink4a^* was conducted as described elsewhere [14].

### Generation of the 2341^luc^ mouse model, bioluminescence imaging and preclinical treatment

2341 cells were infected with lentivirus expressing luciferase (Genecopoeia LPP-FLUC-Lv105-025) and infected cells were selected in the presence of 0.5 μg/mL puromycin. Adding 500 μg/mL luciferin to 1 × 10^6^ cells and measuring bioluminescence verified luciferase expression. For orthotopic tumor growth 6-week old Nod scid gamma (NSG; Jackson Lab) and FVB/N mice (Simonsen Laboratories, Gilroy, CA) were implanted with 30,000 cells/animal and for preclinical testing FVB/N mice were injected with up to 4 × 10^5^ 2341^luc^ cells/mouse as previously described [23]. The protocol was modified by suspending cells in 3 μl Matrigel (Corning Life Sciences). Using a 10 μl Hamilton gas-tight syringe and a stereotactic device (Stoelting; Wood Dale, IL), cells were implanted and the needle was withdrawn 120 seconds after injection. The burr hole was plugged with sterile bone wax and skin closed with Vicryl Rapide (Ethicon). For preclinical testing, at 30 days post-implantation, mice were randomly assigned to treatment, which consisted of vehicle control (DMSO), dabrafenib at 2.5 mg/kg daily by intraperitoneal injections [24], trametinib treatment at 1 mg/kg daily by oral gavage [25] or a combination of both for up to 14 days.

Bioluminescence imaging (BLI) was conducted twice weekly and signal intensity was quantified as the sum of detected photons per second within the region of interest using the Living Image software package and expressed as normalized BLI (fold-change from the start of treatment or original radiance). For survival studies, all animals were kept on treatment until reaching the major endpoint, which was animal neurological symptom free survival. BLI was measured using a Xenogen IVIS Spectrum Imaging System.

### Alamar Blue cell viability assay and agent treatment

Viability assays were conducted as previously described [22] with the modification that 1000 cells per well were plated and Alamar Blue was added at 24 hours and fluorescence was measured 48 hours after plating. To assess dose-inhibition curves and IC50 values, we used concentrations between 0.1 nM and 0.5 μM of vemurafenib, dabrafenib, or trametinib (Selleckchem). Each drug was tested alone or in combination in three replicates in three independent experiments. DMSO-treated cell values were considered as 100% viability. The IC50 concentration was used to treat cells for four or five days at 48 hours intervals with dabrafenib, trametinib, or both before flow cytometry and immunoblotting.

### Flow cytometry

Treated 2341 cells were fixed in 2% paraformaldehyde, permeabilized in 100% ice-cold methanol and stained with rabbit p-ERK antibody (Cell Signaling #4377S) and donkey anti-rabbit IgG-647 (Jackson Labs #711-136-152). Cell cycle analyses were conducted by treating cells with Hoechst 33342 at a final concentration of 2 ng/ml and DNA content (2n versus 4 n) was determined as described [26]. Analyses were conducted using the FACS ARIA II (BD Biosciences). Annexin V staining was performed on unfixed cells using the FITC Apoptosis detection Kit according to manufacturer instructions (BD Cell Analysis).

### Immunoblotting

Treated 2341 cells were stimulated with EGF (20ng/ml) for 20 min prior to protein extraction which was performed using cell lysis buffer (Cell Signaling) supplemented with proteinase and phosSTOP phosphatase inhibitor cocktail (Roche). Proteins were separated by SDS-PAGE and transferred onto polyvinylidene difluoride membranes, which were then probed with primary antibodies, followed by horseradish peroxidase-conjugated secondary antibody, and visualized by ECL (GE Healthcare). Antibodies specific for p-EGFR (Tyr1068), p-Akt (Ser473), total Akt, p-ERK, and beta-actin were from Cell Signaling Technology. Total EGFR and total ERK antibodies were from Santa Cruz Biotechnology, Inc.

### Immunofluorescence (IF)

Tissue sections from 4% paraformaldehyde (PFA)-perfused mouse brains were cut and stained as 10 μm sections. Sections were post-fixed with 4% PFA for 10 minutes, washed and blocked with PBS-0.1% triton-5% normal donkey serum. All sections were incubated with primary antibody and secondary antibody, as well as streptavidin conjugate as described [22].

The following antibodies and streptavidin conjugates were used: Rabbit anti-pERK (Cell Signaling Technology, dilution 1:200), rabbit anti-p-Akt (Ser473, D9E, Cell Signaling Technology, Inc, dilution 1:200), rabbit anti-Gfap (DAKO, dilution 1:400), goat anti-Olig2 (R&D, dilution 1:500), goat anti-Ki67 (Santa Cruz, dilution 1:200), rabbit anti-Ki67 (Cell Signaling Technology, dilution 1:200) and rabbit anti-cleaved caspase-3 (Cell Signaling Technology, dilution 1:400). The secondary antibodies used were donkey IgG-fluorescence conjugates (Jackson ImmunoResearch, dilution 1:400). Images were obtained using a Zeiss AxioImager M1 microscope with Zen 2 software (Carl Zeiss microcopy 2011). Images were also acquired at the 6D high throughput epifluorescent system (Nikon Imaging Center at UCSF) The presence of tumors was confirmed by DAPI staining and H&E staining of consecutive sections.

### Statistics and analysis

Statistical analyses were conducted as indicated in the figure legends using GraphPad Prism 5.0 and Microsoft Excel. For determination of the IC50s *in vitro* drug efficacy studies non-linear regression analyses were utilized. A two-tailed paired t-test was conducted to analyze for significant differences between two treatment groups in flow cytometry experiments assessing DNA content and annexin V. An unpaired t-test was conducted to analyze differences between two treatment groups in flow cytometry experiments detecting p-ERK. One-way ANOVA was used to analyze differences between three or more groups. For Kaplan–Meier survival rate analyses, the number of surviving mice in the three groups of animals were recorded daily after 2341^luc^ implantation. The data were subjected to Log-rank test in order to determine if significant differences existed in survival between the experimental groups. For statistical tests *=p<0.05, **=p<0.01, ***=p<0.001.

## SUPPLEMENTARY FIGURES


