# Supplementary material for: Concurrent MEK targeted therapy prevents MAPK pathway reactivation during BRAFV600E targeted inhibition in a novel syngeneic murine glioma model
Source: Oncotarget. 2016 Oct 3;7(46):75839–53. doi: 10.18632/oncotarget.12419 (PMC5342782; doi:10.18632/oncotarget.12419)
Supplement: Supplementary file 1 [file oncotarget-07-75839-s001.pdf]

Concurrent MEK targeted therapy prevents MAPK pathway reactivation during BRAF^{V600E} targeted inhibition in a novel syngeneic murine glioma model

SUPPLEMENTARY FIGURES

GENE	Mutation location	Forward Primer	Reverse Primer	Status
Akt3	c.1080G>A= p. M360I	TATGGCCGAGCAGTAGACTGG	CCTCCACCAAGGCGTTTATT	wildtype
H3f3a	K27M	GGGTTCTGGCAGGAATACTG	GGTTTCTTCACCCCTCCAGT	wildtype
Hist1h3a	K27M	CTGAACTCACCATCTCCAAGTG	GCCTGTGAGGCCTACCTTGT	wildtype
Kras	c.35G>A = p.G12D	TGCTGAAAATGACTGAGTATAAACTTGTG	CTACCAGGACCATAGGCACATC	wildtype
Map2k1	c.370C>T = p.P124S	AACGGTGGAGTGGTCTTCAAG	GTGCTCCATGCAGATGCTGA	wildtype
Pik3ca	c.1624G>A = p.E542K	GGGTTAGAAGATCTGCTGAACCC	ACTAAGCAGTACATCTGGGCC	wildtype
Pik3r2	1333C>T = p.P445*	CTCAGAGCCCCTTACCTTCT	TGAAGGCCTCTATGGCTGTG	wildtype
Pten	c.388C>G = p.R130G	AGCAGCTTCTGCCATCTCTC	CACACACAGGCAATGGCTGA	wildtype

Supplementary Figure S1: List of oncogenes and tumor suppressors analyzed by targeted sequencing of 2341^{luc} cells. Genes were selected based on their high frequency of mutation in high-grade glioma. We found no mutations in the analyzed genes.

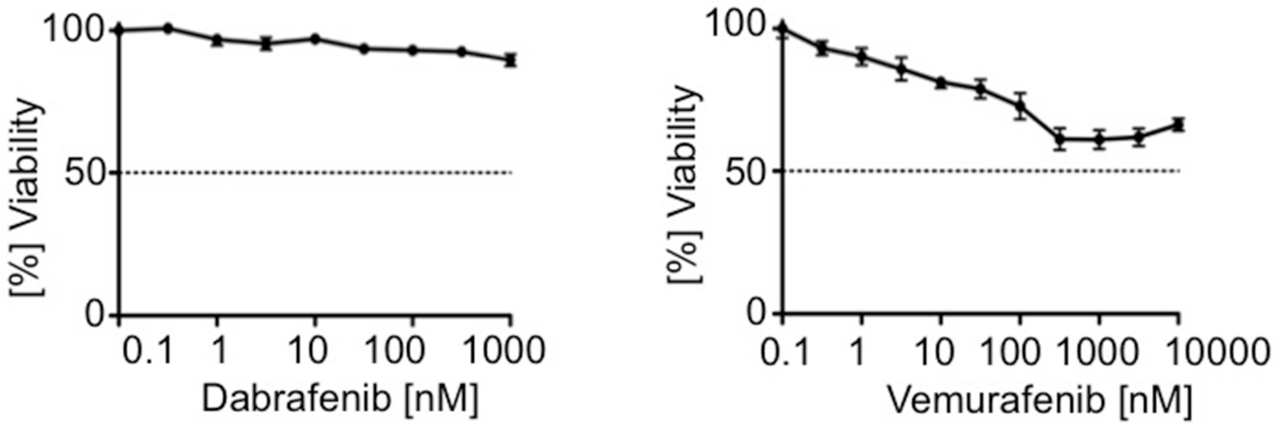

Supplementary Figure S2: Response of BRAF wild type tumor cell line to BRAF^{V600E}-targeted inhibition. Dose-response-curve on BRAF wild-type tumor cell line SF188 showing *in vitro* viability effects of increasing concentrations of dabrafenib (left panel) and vemurafenib (right panel) (n= 3).

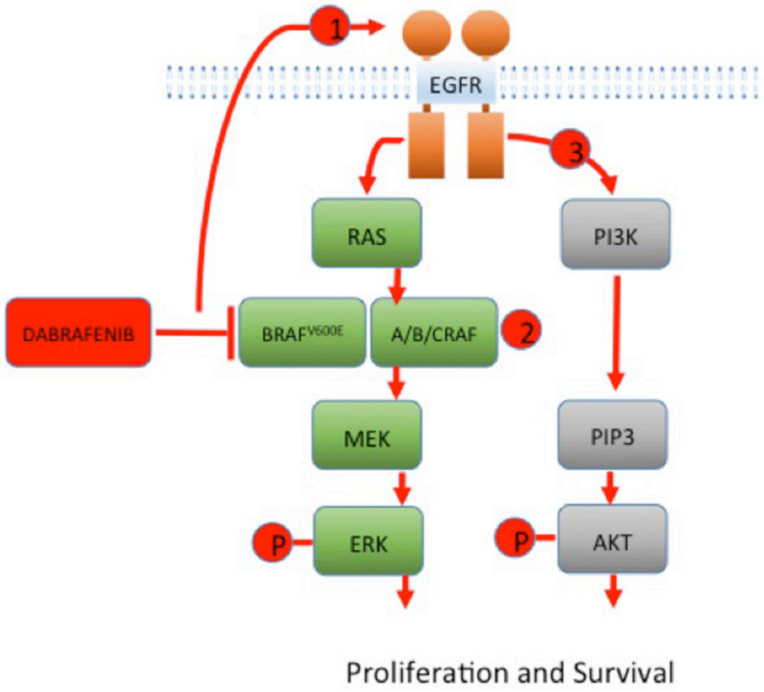

Supplementary Figure S3: Model for adaptive, acquired resistance to dabrafenib in 2341^{luc} model. Resistance to dabrafenib is caused by hyperactivation of MAPK- and PI3k-Akt pathways. Elevated levels of phospho-Erk (pErk) and phospho-Akt (pAkt) following prolonged dabrafenib treatment are potentially achieved upregulation of EGF receptor expression, release from a negative feedback loop (1, 3), by an amplification of BRAF^{V600E} or an alternative Raf kinase activity (2).

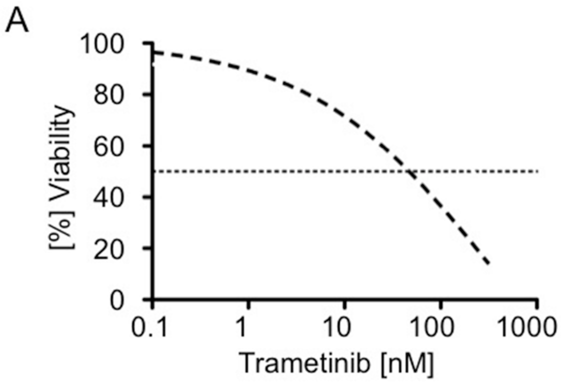

Supplementary Figure S4: Response of 2341^{luc} cells to the MEK inhibitor trametinib. Dose-response-curve showing *in vitro* viability effects of increasing concentrations of MEK inhibitor trametinib on Braf^{V600E} mutant cells. IC50=30.5 nM.

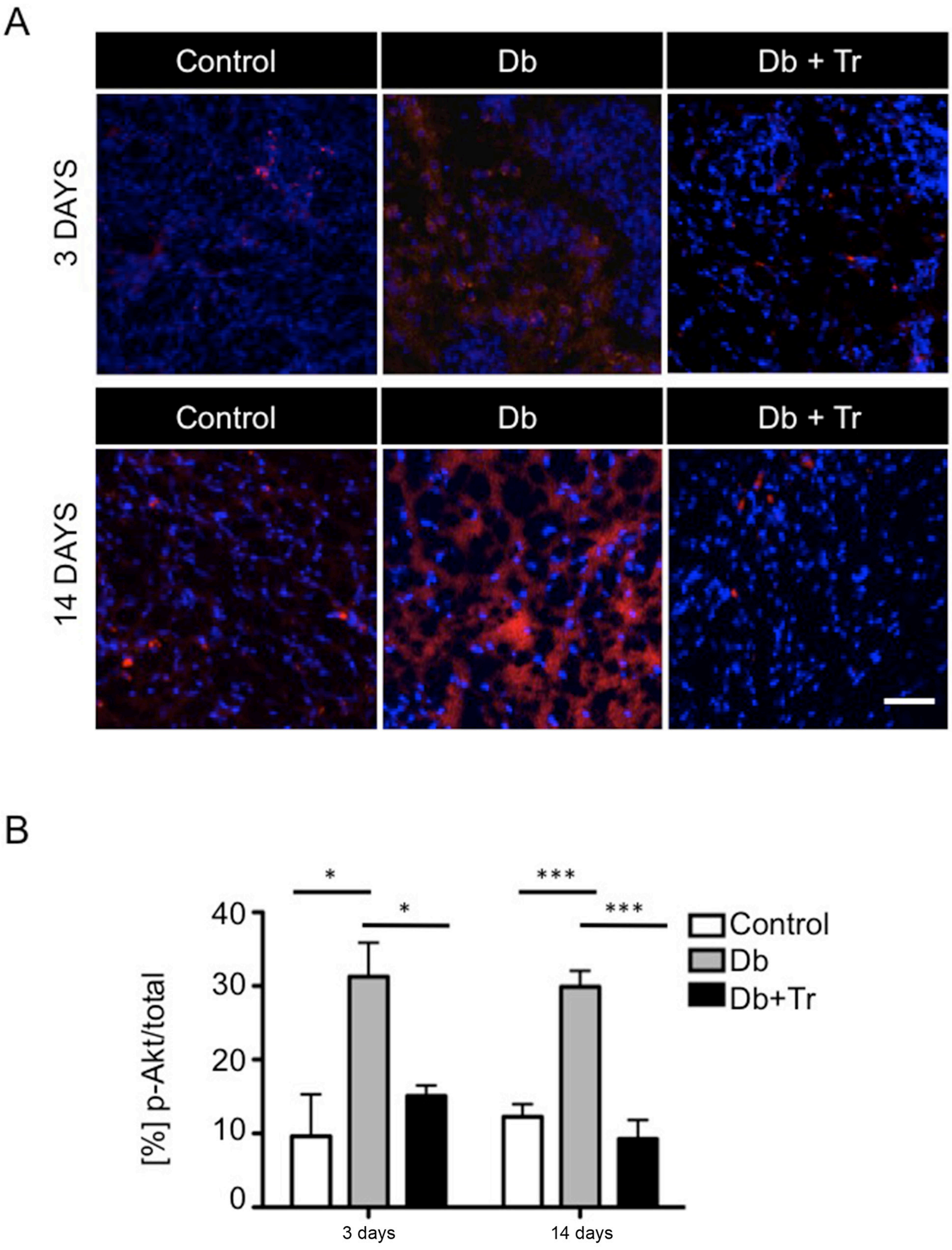

Supplementary Figure S5: Phospho-Akt level increases caused by Db treatment are eliminated by combination therapy. A. Immunofluorescence for phospho-Akt (p-Akt) in 2341[luc] syngeneic graft-bearing mice treated with dimethylsulfoxide (Control), dabrafenib (Db), or a combination of both (Db+Tr), for 3 days and 14 days, respectively, starting at day 30 post-implantation (n=8/group). P-Akt staining is in red. DNA is stained with DAPI and is in blue. Scale bar is 50 µm. **B.** Quantification of p-Akt+ tumor cells in 2341[luc] syngeneic graft-bearing mice treated as described in (A). P-Akt+ cell frequency was determined using Image J. Db monotherapy significantly increased p-Akt+ cell frequency already after 3 days of treatment. The increase was eliminated by combination treatment with Db and Tr (*=p=0.025 for Db versus control; *=p=0.015 for Db versus Db + Tr). P-Akt activity was increased more significantly during the resistant phase, after 14 days or Db treatment, and compared with control (***=p=0.0007) and combination treatment (***=p=0.0009 for Db versus Db + Tr).
